# Experimental Research on Interferometric Inverse Synthetic Aperture Radar Imaging with Multi-Channel Terahertz Radar System

**DOI:** 10.3390/s19102330

**Published:** 2019-05-20

**Authors:** Ye Zhang, Qi Yang, Bin Deng, Yuliang Qin, Hongqiang Wang

**Affiliations:** College of Electronic Science and Technology, National University of Defense Technology, Changsha 410073, China; fighting_zy10@126.com (Y.Z.); dengbin_nudt@163.com (B.D.); qinyuliang@nudt.edu.cn (Y.Q.); oliverwhq@tom.com (H.W.)

**Keywords:** terahertz (THz) radar, interferometric inverse synthetic aperture radar (InISAR), multi-channel radar, THz imaging

## Abstract

The all solid-state terahertz (THz) radar has obvious miniaturized integration and high resolution imaging advantages compared with conventional microwave radar. In this paper, a 0.22 THz active frequency-modulated pulse radar system with one transmission channel and four receiving channels is presented, and interferometric inverse synthetic aperture radar (InISAR) imaging experiments, which can acquire altitude information of objects, are carried out. In order to acquire high-quality InISAR images, a calibration method is presented to solve the nonlinearity of wideband signal frequency and phase inconsistency of different receiving channels together. Furthermore, to deal with the phase wrapping in InISAR imaging of objects with large scale, a novel method based on the dominant scatterers to estimate the objects rotation rate is presented. Finally, to show more information of objects in the InISAR images, the imaging results with a large rotation angle by the convolutional back-projection algorithm are obtained. The imaging results verify the superior performance of the multi-channel THz radar system and the imaging processing method, which can provide support for further research on InISAR imaging in the THz band.

## 1. Introduction

Terahertz (THz) waves lie between microwaves and infrared waves in the electromagnetic spectrum, and they have many special characteristics. Among the numerous applications of THz waves, THz imaging techniques have been receiving sustained attention in recent years due to several exciting advantages of THz waves. Compared to microwaves, with THz radiation it is easier to achieve higher carrier frequency and larger absolute bandwidth, which provide higher spatial resolution and more details of objects. Besides, THz waves can achieve imaging at higher frame rates, and can be used in video synthetic aperture radar (ViSAR) imaging. Compared to infrared waves, THz waves have the ability to penetrate non-metallic and liquid non-polar materials, which makes it possible to realize perspective imaging. In addition, the lower photon energy of THz waves nearly causes no harm to the human body under safety inspection. All these characteristics lead to promising prospects in biomedical imaging [1], stand-off detection for imaging [2,3], and synthetic aperture radar (SAR)/inverse synthetic aperture radar (ISAR) imaging [4,5].

With the noticeable progress of THz sources and detectors over the past few decades, the imaging and recognition with THz radar systems becomes possible. Until now, THz radar imaging systems could be divided into three main categories: raster-scanning radar systems, mixed-scanning radar systems, and SAR/ISAR systems. A raster-scanning radar system uses several lenses to focus the beam onto a fixed area, and the image is obtained by recording the data of each scanning area when scanning along an object. One of the leading institutions in this area is the Jet Propulsion Laboratory (JPL, Pasadena, CA, USA). From 2006 to 2014, they presented serious radar systems with different operating frequencies and bandwidths, and achieved sub-centimeter resolution over a 4–25 m distance [2,6,7,8,9,10]. Another representative institution is the Pacific Northwest National Laboratory (PNNL, WA, USA). In 2009, they presented a standoff, three-dimensional (3-D) imaging prototype that operates near 0.35 THz [3,11]. This system allows screening at ranges of 2–10 m with sub-centimeter resolution, and can obtain an image in 10 s. The imaging frame rate of these systems is determined by the number of scanning pixels and oscillation frequency of the scanning mirrors, so it is time-consuming to acquire a target image. Furthermore, the system structure is complex and easily damaged.

Compared to the raster-scanning radar system, the mixed-scanning radar system substitutes one-dimensional (1-D) raster scanning with 1-D mechanical scanning or 1-D electrical scanning, and the imaging speed can be greatly improved. In 2012, the Chinese Academy of Sciences (CAS, Beijing, China) proposed a 0.22 THz radar system based on the combination of fan-beam scanning and aperture synthesized reconstruction techniques [12,13,14]. The high resolution in the horizontal and vertical direction is achieved by the narrow side of the fan-beam through raster scanning and synthetic aperture through mechanical scanning, respectively. The imaging resolution is about 7 mm in the horizontal direction and better than 4 mm in the vertical direction, and the image reconstruction time is less than 3 s. In 2018, China Academy of Engineering Physics (CAEP, Mianyang, Sichuan) and National University of Defense Technology (NUDT, Changsha, Hunan) presented a 0.34 THz radar system [15,16]. This system incorporates a multiple-input multiple-output (MIMO) array as an electronic beam former in the horizontal dimension and an elliptic cylinder as a focusing reflector in the vertical dimension. The imaging resolution is about 14 mm in the horizontal direction and 12 mm in the vertical direction at a distance of 3 m, and the image reconstruction time is less than 1 s. Although the mixed-scanning radar system can operate near real time, there still exist some defects: (1) the target needs to be stationary; (2) the imaging field of view is limited.

SAR/ISAR systems acquire the target image through relative motion between the radar and target [17,18,19,20]. The resolution depends on the bandwidth of sweep signal and the length or relative rotation angle of synthetic aperture. The Research Institute for High Frequency Physics and Radar Techniques (FGAN-FHR, Dortmund, Germany) has performed serious studies on SAR/ISAR imaging systems in the THz band. In 2007, they presented a 0.22 THz terahertz imaging radar system COBRA-220 which realized a 1.8 cm resolution at a distance of 135 m [21]. In 2013 and 2015, they continuously developed a 0.3 THz system and carried out SAR/ISAR imaging experiments on complex targets such as car and bicycle [4,22]. The system bandwidth is 40 GHz which realizes a 3.75 mm resolution. In 2012, the US Defense Advanced Research Projects Agency (DARPA, Arlington, Virginia, USA) presented the ViSAR program. The program aims to achieve moving targets tracking and imaging with video frame rate. They have carried out the ViSAR experiments with a 0.235 THz one-input four-output system, and obtained the real-time high-resolution SAR image of moving targets [23]. Beside the above two forerunners, other organizations also started to develop SAR/ISAR systems and many remarkable achievements had been made [5,24,25,26,27,28]. Compared to the other THz radar systems, the SAR/ISAR system has a relative simple system structure and low hardware cost, and has no limitation of target distance. However, The SAR/ISAR system can only capture the projected two-dimensional (2-D) characteristics of the target, which lose the altitude information.

In this paper, we present a 0.22 THz all-electronic one-input four-output imaging radar system. The radar system operates in frequency modulated pulse mode with a bandwidth of 5 GHz, and its pulse width, duty circle, and time delay of local oscillator signal can be set flexibly. This design is for the application of connecting traveling-wave tube amplifier to develop long-distance experiments in the future. Compared with the single-channel SAR/ISAR imaging system, our system can achieve 3-D images of objects through interferometry technique [29,30,31], where the altitude information can be obtained from the phase difference of ISAR images of different receiving channels. To verify the system performance, interferometric inverse synthetic aperture radar (InISAR) imaging experiments were carried out, and necessary signal processing methods had been applied to acquire high-quality InISAR images.

The remainder of this paper is organized as follows: Section 2 describes the structure and performance of the multi-channel THz radar system. Section 3 describes the signal model of InISAR imaging. Section 4 presents a serious of signal processing methods of radar echoes. Section 5 gives the experimental results and performance analyses. Finally, Section 6 summarizes this paper.

## 2. 0.22 THz Multi-Channel Radar System

**Figure 1:**
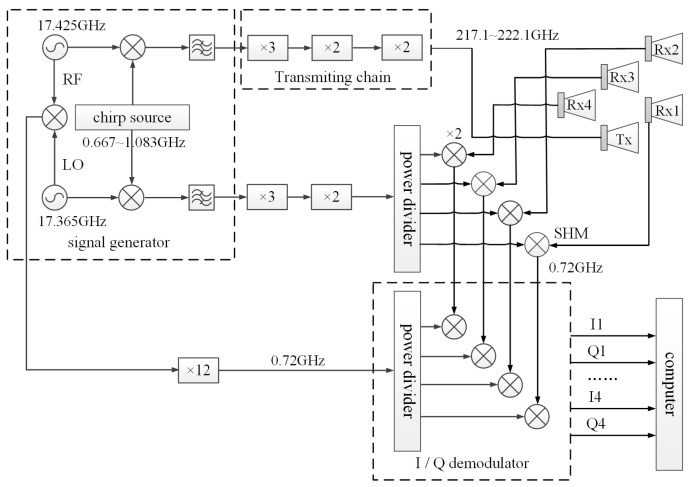
Schematic diagram of the 0.22 THz multi-channel radar system.

The radar system is based on linear frequency modulated (LFM) pulse principle and has a synthetic bandwidth of 5 GHz. The transmitter is a chirped signal from 217.1 GHz to 222.1 GHz, thereby realizing a 3 cm theoretical range resolution. The echo signals are received by four receiving channels simultaneously. The schematic diagram of the radar system is shown in Figure 1. The 0.22 THz multi-channel radar system consists of five modules: the signal generator, radio frequency (RF) front-end, intermediate frequency (IF) processing module, data acquisition module, and cone-shaped horn antennas.

The power loss of the mixer in this radar system is up to 9 dB, one sweep signal source can hardly drive the transmit chain and receive chain together. Besides, the signal bandwidth of THz radar system is so large that it is difficult to directly sample a THz wideband signal using the analog-digital converter, let alone the system has four receiving channels. To solve these problems, the system adopts the super-heterodyne architecture and two sweep signal sources are used to drive the transmit chain and receive chain respectively. This method expands bandwidth of the RF signal by frequency multiplication of a Ku-band signal because it is easy to achieve characteristics of high stability, high power, low phase noise, and low spur. The echo signal to be sampled is transformed to zero IF based on twice mixing processing, which greatly reduces the demand for the sampling rate.

The wideband sweep signal of transmitting end is generated by 12× multiplier up-converting a chirp signal from 0.667 GHz to 1.083 GHz with a fixed frequency signal at 17.425 GHz. The 0.22 THz frequency multiplier is designed based on GaAs planar Schottky diodes, and its structure is very simple. A planar Schottky varactor flip chip with four anodes arranged in anti-series is mounted onto a quartz based microstrip circuit to realize frequency multiplication. The maximum output power of the transmitter is 14 dBm, and the power flatness is within ±1 dB. The 0.22 THz subharmonic mixer (SHM), used for frequency down-converting in Rx chain, is also designed based on low parasitic component Schottky diodes, and suspended microstrip structure is employed. The conversion loss of the mixer is 9 dB. In order to achieve an ideal range resolution, a good linearity of the chirp signal is necessary. The chirp source in our system is achieved by field programmable gate array (FPGA) and digital-analog converter (DAC) with direct digital synthesis technique, which ensures a high linearity with ns-scale hopping time. The two fixed frequency signals at 17.425 GHz and 17.365 GHz are directly achieved by two coherent phase-locked oscillators and a temperature compensated crystal oscillator, and provide reference frequency for the whole radar system. Based on this signal generation method, the center frequency and bandwidth of the chirp source can be changed flexible according to the future requirement. The sweep pulse width can be set from 10 us to 1 ms, and the pulse repetition frequency can be set from 250 Hz to 10,000 Hz.

The echo signals are mixed using a sub-harmonic mixer (SHM) pumped by the local oscillator (LO) signal after power allocation in each channel, and the output of SHM is an IF signal with the center frequency of 0.72 GHz. In order to acquire the baseband signal, the IF signals of four channels are demodulated in four IQ demodulators and sampled by a high-speed ADC. The in-band noise figure and noise temperature are measured as 6–8 dB and 1200–2080 K, respectively, based on the Y factor method. The receiver bandwidth is 20 MHz and the receiver sensitivity is −80 dBm. The insertion loss and isolation of the power divider are 3 dB and 15 dB, respectively.

**Figure 2:**
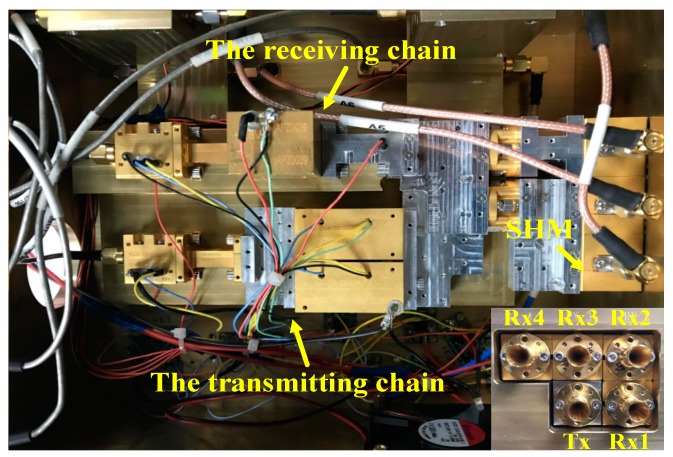
Photograph of front-end setup.

The five antennas are distributed in two rows with three receiving antennas in the upper row, and one transmitting antenna and one receiving antenna in the other row. The gain of the five cone-shaped horn antennas is 25 dB. Rx1 and Rx2 form the vertical interferometric baseline, and the baseline length is 2.1 cm. Rx2 and Rx3 or Rx2 and Rx4 form the horizontal interferometric baseline, and the baseline length is 2.1 cm and 4.2 cm, respectively. In the InISAR imaging experiments, Rx4 is neglected. This array configuration can be flexible changed by connecting external waveguide. The photograph of the front-end setup is shown in Figure 2. Besides InISAR imaging, this system also has many other potential applications such as InSAR imaging, ViSAR imaging, and micro-motion target 3-D imaging.

## 3. Signal Model of InISAR Imaging

**Figure 3:**
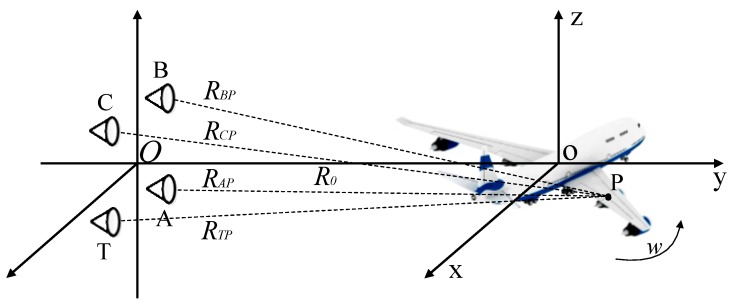
Geometry of InISAR imaging configuration.

After range alignment and auto-focus processing [32,33], the InISAR imaging geometry can be simplified as turntable imaging model, and the geometry is shown in Figure 3. The antenna A, B, and C correspond to the antenna Rx1, Rx2, and Rx3 in Figure 2. Antenna T acts as the transmitter only, and antennas A, B, and C operate at the receiving mode only. A target coordinate system o-xyz is built on the target, where o denotes the center of turntable. $P(x_{P},y_{P},z_{P}$) is an arbitrary scattering point located on the target. $R_{0}$ denotes the vertical distance from coordinate origin o to the antenna plane, *w* denotes the rotation rate of target, and $R_{TP}$, $R_{AP}$, $R_{BP}$ and $R_{CP}$ denote the initial distances from P to four antennas, respectively. The coordinates of T, A, B, and C are $\left(L/2,-R_{0},-L/2\right)$, $\left(-L/2,-R_{0},-L/2\right)$, $\left(-L/2,-R_{0},L/2\right)$ and $\left(L/2,-R_{0},L/2\right)$, respectively. The transmitting LFM pulse from antenna *T* is:

(1) $$s\left(\hat{t},t_{m}\right)=\mathrm{rect}\left(\frac{\hat{t}}{T_{p}}\right)exp[j2\pi \left(f_{c}t+\frac{1}{2}\gamma {\hat{t}}^{2}\right)]$$

where:

(2) $$\mathrm{rect}\left(u\right)=\{\begin{array}{cc} 1 & \left|u\right|\leq 0.5\\ 0 & \left|u\right|>0.5 \end{array}$$

$T_{p}$ is the pulse width, $t_{m}$ is the slow time, $\hat{t}$ is the fast time, $t=t_{m}+\hat{t}$ is the full time, $f_{c}$ is the carrier frequency, and γ is the chirp rate.

Ignore the signal envelope and the received signal at receiver *i* (*i* = A, B, C) from P is:

(3) $$\begin{array}{c} s_{i}\left(\hat{t},t_{m}\right)=\sigma_{P}exp\{j2\pi [f_{c}\left(t-\frac{R_{TP}\left(\hat{t},t_{m}\right)+R_{iP}\left(\hat{t},t_{m}\right)}{c}\right)+\frac{1}{2}\gamma \left(\hat{t}-\frac{R_{TP}\left(\hat{t},t_{m}\right)+R_{iP}\left(\hat{t},t_{m}\right)}{c}{)}^{2}\right]\} \end{array}$$

where *c* is the wave propagation velocity, $\sigma_{P}$ is the reflection coefficient of *P*, and $R_{iP}\left(\hat{t},t_{m}\right)$ represents the distance from receiver *i* to scattering point *P* at time *t*.

The echo signals are processed with a dechirping manner and residual video phase (RVP) correction, and the expression of the radar echo becomes:

(4) $$s_{i}\left(\hat{t},t_{m}\right)=\sigma_{P}exp[-j2\pi \left(\gamma \hat{t}+f_{c}\right)\frac{R_{TP}\left(\hat{t},t_{m}\right)+R_{iP}\left(\hat{t},t_{m}\right)}{c}]$$

For the InISAR imaging, the distance from antennas to the turntable center is far larger than the size of target, thus the assumption of plane wave model is generally applied to simplify the signal expression. In this assumption, the radar echo can be expressed as:

(5) $$\begin{array}{c} s_{i}\left(\hat{t},t_{m}\right)=\sigma_{P}exp[-j\frac{2\pi \left(\gamma \hat{t}+f_{c}\right)}{c}\left(2R_{0}+2x_{p}sin(wt\right)+2y_{p}cos(wt)+R_{iP}\left(\hat{t},t_{m}\right)-R_{BP}\left(\hat{t},t_{m}\right))] \end{array}$$

If the coherent accumulation angle *θ* is small enough to feed the range-Doppler imaging condition [20], the approximations sin(wt)≈wt, cos(wt)≈1, $R_{AP}\left(\hat{t},t_{m}\right)-R_{BP}\left(\hat{t},t_{m}\right)\approx Lz_{P}/R_{0}$, and $R_{CP}\left(\hat{t},t_{m}\right)-R_{BP}\left(\hat{t},t_{m}\right)\approx Lx_{P}/R_{0}$ are applied to further simplify the radar echo expression. Perform keystone transformation and 2-D Fourier transformation on the radar echoes, the target ISAR images of three received channels can be reconstructed as:

(6) $$s_{A}\left(y,f_{a}\right)=A_{P}sinc[\frac{2B}{c}\left(y-y_{P}\right)]sinc[T_{a}\left(f_{a}+\frac{2x_{P}w}{\lambda }\right)]exp[j\left(\varphi_{c}+\varphi_{AB}\right)]$$

(7) $$s_{B}\left(y,f_{a}\right)=A_{P}sinc[\frac{2B}{c}\left(y-y_{P}\right)]sinc[T_{a}\left(f_{a}+\frac{2x_{P}w}{\lambda }\right)]exp(j\varphi_{c})$$

(8) $$s_{C}\left(y,f_{a}\right)=A_{P}sinc[\frac{2B}{c}\left(y-y_{P}\right)]sinc[T_{a}\left(f_{a}+\frac{2x_{P}w}{\lambda }\right)]exp[j\left(\varphi_{c}+\varphi_{CB}\right)]$$

where $A_{P}$ is the scattering intensity of scattering point P in the ISAR images, $T_{a}$ is the data acquisition time, *B* is the radar bandwidth, $\lambda =c/f_{c}$ is radar wavelength. $\varphi_{c}$ and $\varphi_{iB}$ (i=A,C) denote the constant phase and interferometric phase, and they are expressed as:

(9) $$\varphi_{c}=-\frac{2\pi }{\lambda }\left(R_{0}+y_{P}\right)$$

(10) $$\varphi_{AB}=-\frac{2\pi Lz_{P}}{\lambda R_{0}}$$

(11) $$\varphi_{CB}=-\frac{2\pi Lx_{P}}{\lambda R_{0}}$$

The above formulas show that the target coordinates can be calculated through Equations (6)–(8), (10) and (11). To avoid the phase wrapping, the absolute value of $\varphi_{AB}$ and $\varphi_{CB}$ should be less than *π*, and we can calculate that the unambiguous size of the target is:

(12) $$\left|x_{P}\right|,\left|z_{P}\right|<\frac{\lambda R_{0}}{2L}$$

## 4. Signal Processing Method

### 4.1. Compensation of Signal Nonlinearity and Phase Inconsistency

In an ideal condition, the frequency of the transmitted wideband sweep signal varies linearly, and the stationary phases of three received signals are identical. Thus, the range resolution will reach the theoretical value c/2B, and the coordinates of the InISAR images accord with the real position of target. In practice, the conversion efficiencies of the high-frequency power amplifiers and multipliers in the transmitting and receiving chains are not flat across the radar bandwidth, which will result in the disturbances of the amplitude and phase in the terahertz wideband signals, and the chirp rate is no longer a constant. This phenomenon will seriously deteriorate the range resolution, even lead to the appearance of harmonics. Moreover, the stationary phases of radar echoes received by different receiving channels are inconsistent because they transmit through different antennas and electrical connecters, and the phase modulation characteristics of these devices are different. It will destroy the real phase relation of the ISAR images of different receiving channels, and make the InISAR imaging results no longer match the real position of target. Take these non-ideal factors into consideration, the radar echo and LO signal can be expressed as:

(13) $$s_{i}\left(t\right)=A_{RF}\left(t-\tau \right)exp(j2\pi \left(f_{c}\left(t-\tau \right)+\frac{1}{2}\gamma \left(t-\tau {)}^{2}+\xi_{RF}\left(t-\tau \right)+\phi_{i}\right)\right)$$

(14) $$s_{LO}\left(t\right)=A_{LO}\left(t\right)exp(j2\pi \left(f_{c}t+\frac{1}{2}\gamma t^{2}+\xi_{LO}\left(t\right)\right))$$

where $A_{RF}\left(t\right)$ is the RF signal amplitude, $A_{LO}\left(t\right)$ is the LO signal amplitude, $\xi_{RF}\left(t\right)$ is the phase-error function, $\phi_{i}$ (*i* = A, B, C) is the initial stationary phase, and τ is the round trip time.

After the mixing processing and RVP correction, the IF signal is described as:

(15) $$s_{iIF}\left(t\right)=A_{RF}\left(t-\tau \right)A_{LO}\left(t\right)exp(-j2\pi \left(f_{c}\tau +\gamma t\tau +\xi_{LO}\left(t\right)-\xi_{RF}\left(t-\tau \right)-\phi_{i}\right))$$

In the THz radar system, the nonlinearities of $\xi_{LO}\left(t\right)$ and $\xi_{RF}\left(t\right)$ are not the same, and the phase term $\xi_{LO}\left(t\right)-\xi_{RF}\left(t-\tau \right)$ will deteriorate the system performance even if the time delay τ is small enough to be neglected. The existence of $\phi_{i}$ brings an error to the interferometric phase in Equations (10) and (11), which will lead to coordinates location error even phase wrapping in the InISAR imaging results.

To compensate the frequency nonlinearity and phase inconsistency of the multi-channel THz radar system, the phase term $\xi_{LO}\left(t\right)-\xi_{RF}\left(t-\tau \right)-\phi_{i}$ should be removed. In this paper, we use a reference signal reflected by a corner reflector located at coordinate origin o to compensate the non-ideal factors. The reference signal is expressed as:

(16) $$s_{iref}\left(t\right)=A_{RF}\left(t-\tau_{ref}\right)A_{LO}\left(t\right)exp(-j2\pi \left(f_{c}\tau_{ref}+\gamma t\tau_{ref}+\xi_{LO}\left(t\right)-\xi_{RF}\left(t-\tau_{ref}\right)-\phi_{i}\right))$$

where $\tau_{ref}=2\sqrt{R_{0}^{2}+L^{2}/2}/c$ is the round trip time of the reference signal. The $\tau_{ref}$ in the three received channels should be the same, otherwise it will bring extra inconsistent phases. This is the reason that the reference target should be located at coordinate origin. The compensated echo signal is expressed as:

(17) $$\begin{array}{cc} s_{iIF\_new}=\frac{s_{iIF}}{s_{iref}} & =\frac{A_{RF}\left(t-\tau \right)}{A_{RF}\left(t-\tau_{ref}\right)}exp\{-j2\pi [f_{c}\left(\tau -\tau_{ref}\right)+\gamma t\left(\tau -\tau_{ref}\right)\\ & +\xi_{RF}\left(t-\tau_{ref}\right)-\xi_{RF}\left(t-\tau \right)]\} \end{array}$$

From Equation (17), we can find that the phase-error function $\xi_{LO}\left(t\right)$ and stationary phase error $\phi_{i}$ are completely removed. Under the real application, the size of the target is limited which make the signals $\xi_{RF}\left(t-\tau_{ref}\right)$ and $\xi_{RF}\left(t-\tau \right)$ have a strong correlation. For example, if the maximum size of a target is 100 m, we can calculate that $\left|\tau -\tau_{ref}\right|$ is less than 0.33 us which can be neglected compared to the signal pulse width. Thus, the residual modulation in amplitude and phase nearly has no effect on the system performance, and the theoretical range resolution and the real position of target can be achieved.

### 4.2. InISAR Image Scaling

We have illustrated that the absolute value of $\varphi_{AB}$ and $\varphi_{CB}$ in Equations (10) and (11) should be less than π to avoid phase wrapping. However, the phase wrapping phenomenon often occurs in practical applications. For example, the baseline length, target distance, and maximum target size are 2.1 cm, 4.1 m, and 52 cm respectively in our experiment, then we can calculate that the maximum absolute value of interferometric phase is larger than π (6.14). Thus, it is necessary to perform phase unwrapping operation before InISAR imaging. In the earlier literatures, some one-dimensional (1-D) and two-dimensional (2-D) phase unwrapping methods applied to InSAR and InISAR have been proposed, such as 1-D path integral method and 2-D fast transforms and iterative methods [34,35,36]. However, the inteferometric phases of the weak scatterers in ISAR images are easily contaminated by noise in these methods, and it is difficult to achieve phase unwrapping operation.

Unlike SAR image, in most cases, a high-frequency small-rotation angle ISAR image of a moving target usually consists of several dominating reflectors, such as corner reflectors formed by the tail, fuselage, and wings of an aircraft. The pixels of these dominating scatterers have stronger SNR and more accurate interferometric phase value than the weak ones. In this paper, we achieve the cross-range scaling of all scatterers based on the phases of these dominating scatterers to estimate the rotation rate of target rather than the conventional phase unwrapping operation. Once the rotational rate is obtained, the cross-range coordinates can be determined directly based on the cross-range-Doppler relationship.

The details of the proposed InISAR image scaling method are described as follows:

Step (1) Extract the dominant scatterers from $s_{B}\left(y,f_{a}\right)$ and $s_{C}\left(y,f_{a}\right)$, and the inseparable dominant scatterers in range-Doppler domain are eliminated using [20]:

(18) $$\Delta V=\left|\frac{\left|s_{B}\left(y,f_{a}\right)\right|-\left|s_{C}\left(y,f_{a}\right)\right|}{\left|s_{B}\left(y,f_{a}\right)\right|+\left|s_{C}\left(y,f_{a}\right)\right|}\right|>0.15$$

Step (2) Extract the interferometric phases of the separable dominant scatterers, and arrange them in the order of Doppler frequency. The 1-D path integral method is applied to achieve phase unwrapping, and the theory is:

(19) $$\varphi_{new}\left(1\right)=\varphi \left(1\right),\;\varphi_{new}\left(k+1\right)=\varphi_{new}\left(k\right)+\Delta \varphi \left(k\right)$$

where:

(20) $$\Delta \varphi \left(k\right)=\{\begin{array}{ll} \varphi \left(k+1\right)-\varphi \left(k\right) & \left|\varphi \left(k+1\right)-\varphi \left(k\right)\right|<\pi \\ \varphi \left(k+1\right)-\varphi \left(k\right)+2\pi & \varphi \left(k+1\right)-\varphi \left(k\right)<-\pi \\ \varphi \left(k+1\right)-\varphi \left(k\right)-2\pi & \varphi \left(k+1\right)-\varphi \left(k\right)>\pi \end{array}$$

φ(k) and $\varphi_{\mathrm{new}}\left(k\right)$ are phases before and after phase unwrapping, respectively. k=1,2,…,K−1 and K is the total number of the dominant scatterers.

Step (3) The cross-range coordinates $x_{k}$ of the dominant scatterers can be obtained through (11) using the $\varphi_{new}\left(k\right)$.

Step (4) From (6)–(8), we can find that there is a linear relationship between Doppler frequency $f_{a}$ and cross-range coordinate x, and it can be expressed as:

(21) $$f_{a}=\frac{-2wx}{\lambda }+\Delta f$$

where Δf denotes the Doppler error. For the K dominant scatterers, the relationship between $f_{a}$ and x is established as:

(22) F=WX

where $F=\left[f_{a1}\;f_{a2}\;\ldots \;f_{aK}\right]$, $X=\left[\begin{array}{cccc} -\frac{2}{\lambda }x_{1} & -\frac{2}{\lambda }x_{2} & \ldots & -\frac{2}{\lambda }x_{K}\\ 1 & 1 & \ldots & 1 \end{array}\right]$, and W=[w Δf]. Therefore, the least mean square (LMS) can be utilized to estimate the rotational rate of target is

(23) $$\hat{W}=FX^{T}{\left(XX^{T}\right)}^{-1}$$

Step (5) Achieve cross-range image scaling based on the estimated rotational rate.

### 4.3. Large-Rotation Angle InISAR Imaging

The small-rotation angle (SA) ISAR images usually lose some target information due to the anisotropic scattering. To obtain high-quality InISAR images, reflected signal with a relative large range of rotation angle are needed. In large-rotation angle (LA) ISAR imaging, the RD algorithm is no longer applied, and convolutional back-projection (CBP) algorithm is adapt [37]. The cross-range scaling can be achieved based on the estimated rotational rate, thus we just need to perform interferometric operation along height direction in LA InISAR imaging.

We have illustrated that a corner reflector located at coordinate origin is adapt as a reference target to compensate the signal frequency nonlinearity and phase inconsistency. Under the geometry in Figure 3, the echo signal with a large angle of a complex target can be expressed as:

(24) $$s_{A}\left(k,\theta \right)=\iint_{x,y}f\left(x,y,z\right)exp(-j2k\left(x\;cos(\theta \right)+y\;sin(\theta )+\frac{Lz}{2R_{0}}))dxdy$$

(25) $$s_{B}\left(k,\theta \right)=\iint_{x,y}f\left(x,y,z\right)exp(-j2k\left(x\;cos(\theta \right)+y\;sin(\theta )))dxdy$$

where $k=2\pi \left(\gamma \hat{t}+f_{c}\right)/c$ is wavenumber, θ=wt is sample angle. The imaging process is expressed as:

(26) $$F_{i}\left(x,y,z\right)=\int_{k_{min}}^{k_{max}}\int_{-\theta_{t}/2}^{\theta_{t}/2}ks_{i}\left(k,\theta \right)exp(j2k\left(x\;cos(\theta \right)+y\;sin(\theta ))dxdy\left(i=A,B\right)$$

where:

(27) $$F_{A}\left(x,y,z\right)/F_{B}\left(x,y,z\right)=exp(-j2\pi \frac{Lz}{\lambda R_{0}})$$

Then the height coordinates can be directly achieved through interferometric operation.

## 5. Experimental Results

### 5.1. Signal Calibration

**Figure 4:**
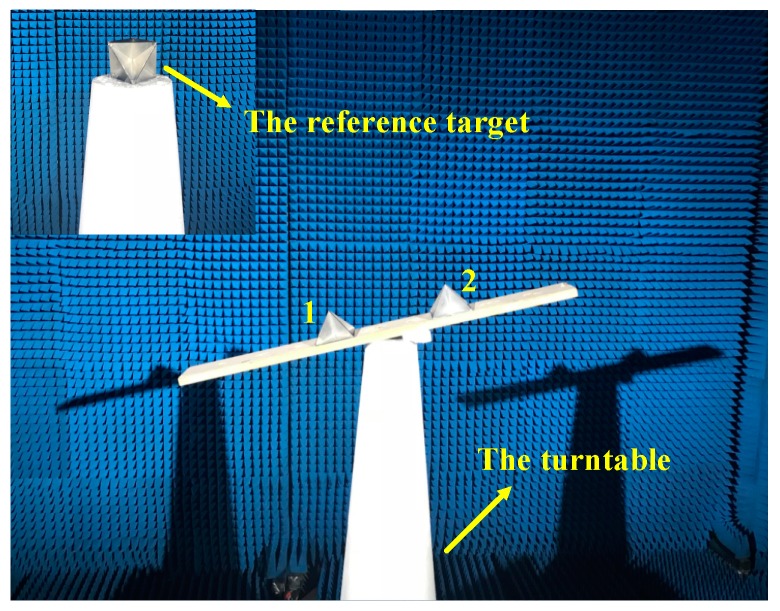
Imaging scene of two corner reflectors.

To test the feasibility of signal calibration method and resolution of the multi-channel THz radar system, an InISAR imaging experiment on two corner reflectors was carried out. The experimental scene are shown in Figure 4. In the experiment, the distance from antenna plane and turntable center is 4.1 m, the altitude difference of the two corner reflectors is about 7.2 cm, the pulse width is 160 μs, the pulse repetition frequency is 2500 Hz, the sample frequency is 12.5 MHz, the rotational rate of turntable is 90°/s, and the total rotation angle is 4°.

**Figure 5:**
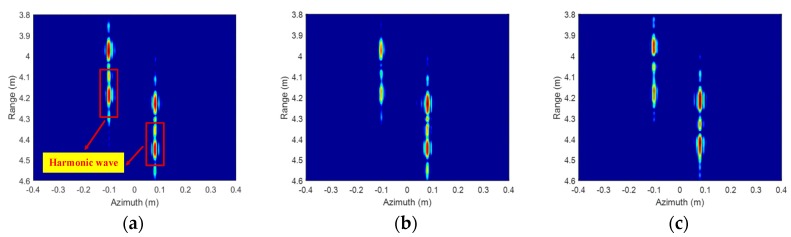
ISAR imaging results of two corner reflectors before signal calibration. (**a**) Antenna A; (**b**) Antenna B; (**c**) Antenna C.

**Figure 6:**
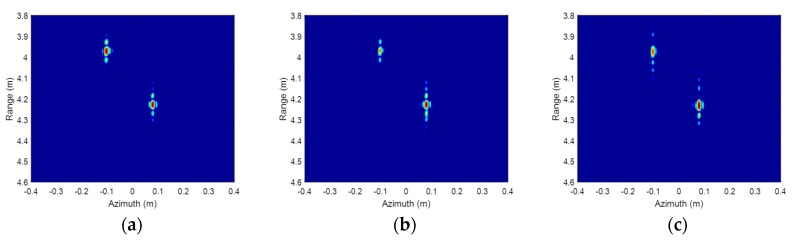
ISAR imaging results of two corner reflectors after signal calibration. (**a**) Antenna A; (**b**) Antenna B; (**c**) Antenna C.

**Figure 7:**
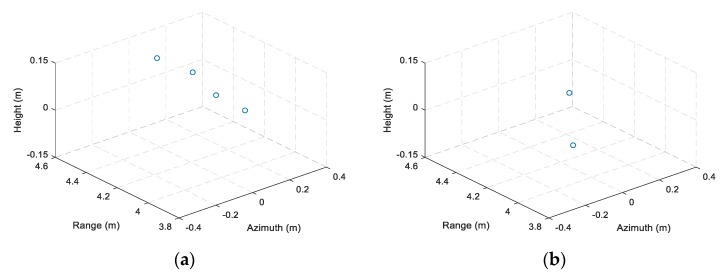
InISAR imaging results of two corner reflectors. (**a**) Before signal calibration; (**b**) After signal calibration.

Figure 5 shows the ISAR imaging results before signal calibration. Due to the influences of wideband signal nonlinearity, the main lobes in range of the corner reflectors are broadened severely, and harmonic waves, which nearly cannot be distinguished from the real target, emerge. Figure 6 shows the ISAR imaging results after signal calibration. The calibration data are radar echoes of a static corner reflector located at the center of the turntable. The signal calibration is achieved by Equation (17) with the radar echoes of the imaging target and reference target. The compensated ISAR images of the two corner reflectors focus well, and the harmonic waves are eliminated. All the ISAR images are displayed in log-magnitude and the dynamic range is 20 dB. The InISAR imaging results before and after signal calibration are shown as Figure 7. The real coordinates and interferometric coordinates in x-axis and z-axis of the two corner reflectors are given in Table 1. The real x coordinates can be obtained from the ISAR images in Figure 6, and the real z coordinates are measured based on the geometric relationship between reference target and imaging target. Compared to the real coordinates, both the x coordinates and z coordinates of the two corner reflectors have a large offset before signal calibration, and the x coordinate of the first corner reflector even encounters phase wrapping. After phase inconsistency compensation, the coordinates in the InISAR imaging result are very close to the real coordinates of target. The above experimental results have fully validated our signal calibration method to compensate the frequency nonlinearity and phase inconsistency. All the experimental results of this paper are obtained on a desktop computer with Intel core i7-7820X 3.60 GHz CPU and 32 GB RAM using Matlab codes. The algorithm reconstruction time, which excludes data import time, of this InISAR imaging experiment is 0.622 s.

**Figure 8:**
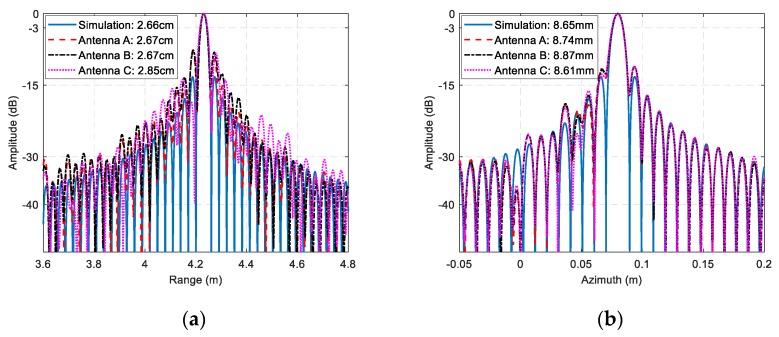
Comparison of range profiles and azimuth profiles between the right corner reflector and an ideal point target. (**a**) Range profiles; (**b**) Azimuth profiles.

To show the resolution of the multi-channel THz radar system, the range profiles and azimuth profiles of the right corner reflector in three ISAR images and simulation results of an ideal point target under the same system parameters are shown in Figure 8. It can be seen that the theoretical range resolution and azimuth resolution are 2.66 cm and 8.65 mm with no use of data window, respectively. The actual range resolutions of three received channels are 2.67 cm, 2.67 cm, and 2.85 cm, and the corresponding azimuth resolutions are 8.74 mm, 8.87 mm and 8.61 mm respectively, which are very close to the theoretical value.

### 5.2. InISAR Imaging Results of Complex Target

**Figure 9:**
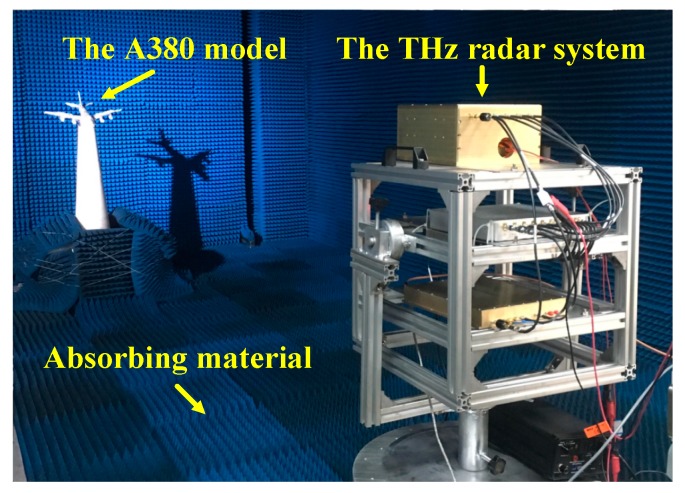
Experimental scenario of an A380 model.

To verify InISAR imaging of complex targets, an experiment on an Airbus A380 model was carried out. The length, wingspan and height of the airplane model are 45 cm, 52 cm and 17 cm, respectively. The experimental scenario is shown as Figure 9. In the experiments, the A380 model was located on the low scattering bracket, and had a pitch angle of about 30°. The experimental parameters are identical to the previous experiment. Considering the anisotropy of the complex target, reflected signals with a large range of rotation angle were collected to achieve high-quality InISAR images.

**Figure 10:**
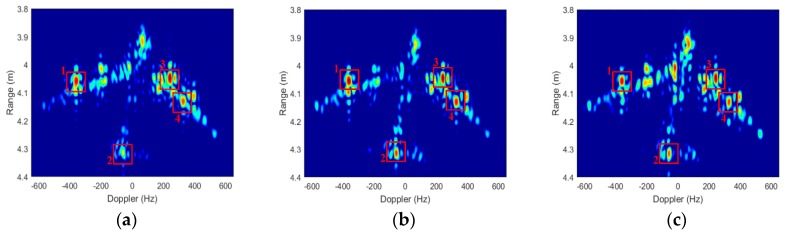
ISAR imaging results of A380 model with small rotation angle. (**a**) Antenna A; (**b**) Antenna B; (**c**) Antenna C.

**Figure 11:**
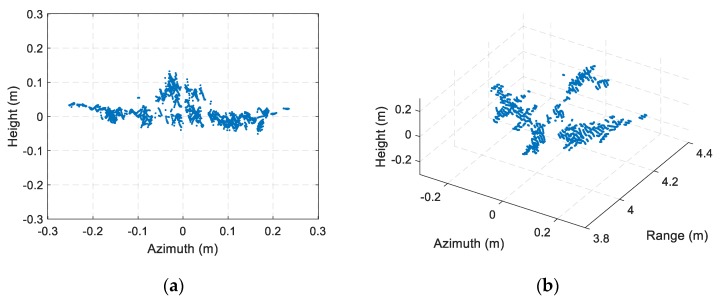
InISAR imaging results of A380 model with small rotation angle. (**a**) Front view; (**b**) Three-dimensional view.

The ISAR imaging results after signal calibration with a small rotation angle (about 4°) of three received channels are shown as Figure 10. All the ISAR images are displayed in log-magnitude and the dynamic range is 25 dB. We can observe that the ISAR images focus well. The coordinate in cross-range is Doppler frequency rather than the target location, because we assume the rotational rate is unknown, which matches most of InISAR real applications. Based on the experimental parameters, we can calculate that the unambiguous size of the target is 26.6 cm. Thus, there exists phase wrapping in cross-range direction, and it is necessary to be solved before cross-range interferometric processing. To achieve image scaling in cross-range direction, the image scaling method described in Section 4.2 is adapt. Four dominant scatterers stronger than 6 dB indicated in Figure 10 are extracted, and the cross-range Doppler frequencies, interferometric phases, and unwrapped cross-range coordinates of these dominant scatterers are given in Table 2. Using Equation (23), we can estimate that the rotation rate is 89.9°/s, which is nearly the same as the real rotation rate 90°/s of the turntable. The estimated rotation rate has verified the effectiveness of the InISAR image scaling method proposed in this paper. The final InISAR imaging results with small rotation angle are shown as Figure 11. The algorithm reconstruction time of this InISAR imaging experiment with 4° rotation angle is 0.784 s. In Figure 11, The SA InISAR images lose part of target information due to the anisotropic scattering. Besides, the interferometric coordinates of the weak scatterers in the ISAR images exist large errors.

**Figure 12:**
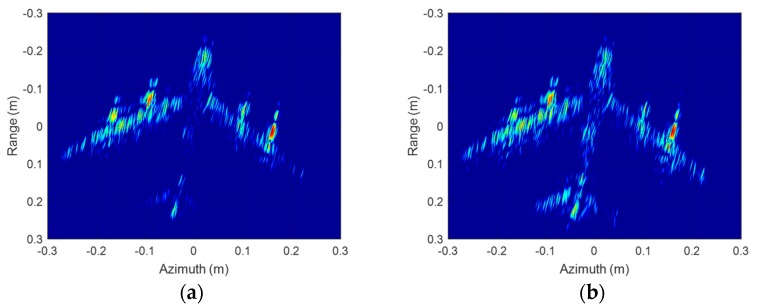
ISAR imaging results of A380 model with 20° rotation angle. (**a**) Antenna A; (**b**) Antenna B.

**Figure 13:**
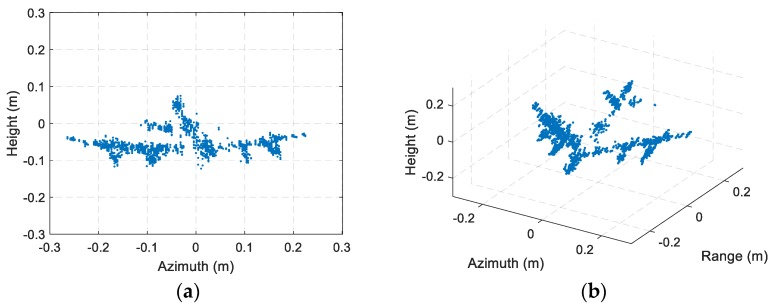
InISAR imaging results of A380 model with 20° rotation angle. (**a**) Front view; (**b**) Three-dimensional view.

To overcome the drawbacks in SA InISAR imaging, radar echoes with a relative large rotation angle are needed. Figure 12 shows the ISAR imaging results with 20° rotation angle. The ISAR images are displayed in log-magnitude and the dynamic range is 25 dB. Since the rotation rate of target has been estimated, the CBP algorithm is directly adapt to produce the ISAR images. Compared to Figure 10, the detailed information of the airplane model is more abundant, the scattering intensity is more uniform, and the resolution in cross-range is higher in Figure 12. The final InISAR imaging results with 20° rotation angle are shown as Figure 13. Comparing with the SA InISAR imaging results, the LA InISAR imaging results are closer to the real imaging target. The key parts in the A380 model such as engine, wing and vertical fin can be clearly identified, which can greatly enhance the probability of target recognition. The algorithm reconstruction time of this InISAR imaging experiment with 20° rotation angle is 3.266 s.

## 6. Conclusions

A 0.22 THz multi-channel imaging radar system with interferometric inverse aperture synthesis technique was developed in this paper. The system structure and its potential applications were detailed presented and discussed. To overcome the non-ideal factors in the radar system, an effective calibration method was presented to obtain a successful image. Besides, an InISAR image scaling method was proposed to solve the phase wrapping problem, and the target rotation rate, which is essential in LA InISAR imaging, was estimated in the meantime. The imaging performance of the radar system and the validity of the imaging processing method were verified experimentally. The imaging results showed that the radar system can achieve high-quality 3-D imaging of complex targets with centimeter-scale resolution in range and azimuth. Until now, all experiments were carried out in laboratory environment, but the work in this paper can provide support to the remote application of terahertz InISAR imaging systems in the future.

## Figures and Tables

**Table 1:** Comparison of target coordinates.

Coordinate (m)	Real Coordinate	Before Signal Calibration	After Signal Calibration
Corner reflector 1	(−0.101, −0.025)	(0.123, 0.039)	(−0.106, −0.025)
Corner reflector 2	(0.082, 0.045)	(0.061,0.109)	(0.082, 0.044)

**Table 2:** Doppler frequencies and coordinates of four dominant scatterers.

Scatterer Number	1	2	3	4
Doppler frequency (Hz)	−357.8	−53.92	250	338.2
Interferometric phase (rad)	2.792	−0.505	2.229	−3.042
Coordinate (cm)	−15.88	−2.30	10.14	14.74
